# Postprandial Differences in the Amino Acid and Biogenic Amines Profiles of Impaired Fasting Glucose Individuals after Intake of Highland Barley

**DOI:** 10.3390/nu7075238

**Published:** 2015-07-09

**Authors:** Liyan Liu, Xinyang Wang, Ying Li, Changhao Sun

**Affiliations:** Department of Nutrition and Food Hygiene, Public Health College, Harbin Medical University, 157 Baojian Road, Nangang District, Harbin 150086, China; E-Mails: yanziliu2100@163.com (L.L.); xiaoxiao455846353@163.com (X.W.)

**Keywords:** postprandial status, amino acid and biogenic amine profiles, insulin, highland barley, impaired fasting glucose

## Abstract

The aim of this study was to measure the postprandial changes in amino acid and biogenic amine profiles in individuals with impaired fasting glucose (IFG) and to investigate the changes of postprandial amino acid and biogenic amine profiles after a meal of highland barley (HB). Firstly, 50 IFG and 50 healthy individuals were recruited for the measurement of 2 h postprandial changes of amino acid and biogenic amine profiles after a glucose load. Secondly, IFG individuals received three different loads: Glucose (GL), white rice (WR) and HB. Amino acid and biogenic amine profiles, glucose and insulin were assayed at time zero and 30, 60, 90 and 120 min after the test load. The results showed fasting and postprandial amino acid and biogenic amine profiles were different between the IFG group and the controls. The level of most amino acids and their metabolites decreased after an oral glucose tolerance test, while the postprandial level of γ-aminobutyric acid (GABA) increased significantly in IFG individuals. After three different test loads, the area under the curve for glucose, insulin, lysine and GABA after a HB load decreased significantly compared to GL and WR loads. Furthermore, the postprandial changes in the level of GABA between time zero and 120 min during a HB load were associated positively with 2 h glucose and fasting insulin secretion in the IFG individuals. Thus, the HB load produced low postprandial glucose and insulin responses, which induced changes in amino acid and biogenic amine profiles and improved insulin sensitivity.

## 1. Introduction

Impaired fasting glucose (IFG) was introduced as a new category of glucose tolerance by the American Diabetes Association and the World Health Organization in order to identify early diabetes [1]. In the USA, the prevalence of IFG was 26.0% in adults [2] and 13.1% in adolescents [3], whereas the prevalence was 0.9%–7.1% in China and Japan [4]. IFG is a state in glucose metabolism intermediate between normal glucose tolerance and type 2 diabetes (T2D) [5]. IFG individuals are characterized primarily by insulin resistance (IR), impaired insulin sensitivity and impaired beta cell function [4,5,6].

Recent research suggested amino acids might be important in the development of IR with regard to alterations in circulating levels of several amino acids, including aromatic amino acids (AAA) and branched-chain amino acids (BCAA) [7,8]. Baseline levels of AAAs and BCAAs are prognostic for improvement in insulin sensitivity in response to dietary/behavioral intervention [9] and other types of amino acids might be relevant in the development of IR and T2D [10,11]. Most of these studies have been done in the fasting state but it is important to note the body is in the postprandial state for most of the day. Changes in metabolism in the postprandial state might contribute to alteration of the physiological functions of the body. It is necessary, therefore, to study the potential effect of metabolic changes of amino acid and biogenic amine profiles in the postprandial state, which will be helpful in exploring the relationship between the postprandial amino acid and biogenic amine profiles and IR.

Time-dependent variations in the hormonal and metabolic profiles in the postprandial state are of great importance to human health. An important change in the postprandial state is acute hyperglycemia following a glucose load or a meal, which leads to metabolic changes, including the levels of insulin, fatty acids, and amino acids *etc.* An oral glucose tolerance test (OGTT) or different meals are usually used to investigate these postprandial time-dependent variations. There have been many human studies using an OGTT to investigate the postprandial change of metabolic profiles in diabetic and IR individuals [12,13]. Bondia-Pons *et al.* reported that rye bread and white wheat bread have an effect on postprandial metabolic profiles in healthy individuals [14]. Therefore, it is necessary to investigate systemically the effect of equivalent loads of glucose in the form of highland barley (HB) and white rice (WR) on the postprandial amino acid and biogenic amine profiles in IFG individuals.

The objectives of this study were: (1) to investigate the difference of postprandial amino acid and biogenic amine profiles between IFG and healthy individuals using an OGTT and a targeted metabolomics approach; and (2) to evaluate the effect of a HB load on amino acid and biogenic amine profiles in IFG individuals and to explore the association of these postprandial profiles with IR, thereby opening new perspectives in the study of the postprandial physiological reaction of IFG individuals following glucose ingestion or an HB load.

## 2. Experimental Section 

This study was approved by the Ethics Committee of Harbin Medical University, China. The study was conducted in accordance with the Declaration of Helsinki. Written informed consent was obtained from each participant. The clinical register number was ChiCTR-TRC-12002630.

All participants were recruited in 2013 from the Hexing and Yixing Districts in Harbin City, Heilongjiang Province in northern China via posters in the Districts. The glycemic state was classified according to the American Diabetes Association criteria [15] after a 75 g OGTT. Participants with fasting plasma glucose (FPG) levels between 6.1 and 6.9 mmoL/L and a 2 h post challenge glucose (2h-PG) level of <7.8 mmoL/L were identified as IFG. The exclusion criteria were: any cardiovascular complication or inflammatory disease, and any medications such as antioxidants and lipid-lowering or glucose-lowering drugs. The design of this study is illustrated in Supplementary Figure S1.

Data for age, weight, height, alcohol use, cigarette smoking, menstrual status, as well as physical activity at work and at leisure was obtained from questionnaires. Dietary intakes were estimated with a validated semi-quantitative food frequency questionnaire.

### 2.1. Study 1: Amino Acid and Biogenic Amine Profiles Change in IFG Individuals during 120 min OGTT

IFG (50) and control participants (50) were recruited according to FPG and 2h-PG levels. There was no significant difference (*p* > 0.05) between the two groups with regard to daily physical activity level (data not shown), age, gender, cigarette smoking, alcohol consumption or dietary intake (Table 1). Body mass index (BMI), systolic blood pressure, diastolic blood pressure, levels of triglycerides (TGs) and total cholesterol (TC), FPG, 2h-PG and hemoglobin A1c (HbA_1c_%) were significantly different (*p* < 0.05) between IFG individuals and controls (Table 1).

After 12 h fasting, blood samples were collected and participants were challenged with the equivalent of 75 g anhydrous glucose dissolved in 250 mL water. No food or drink was consumed during the test. After 2 h, post-challenge blood samples were collected, separated by centrifugation (3000× *g* for 15 min) at room temperature and the supernatant was stored at –80 °C.

**Table 1:** Demographic and Clinical Chemistry Characteristics of Human Subjects.

Parameter	Control (*n* = 50)	IFG (*n* = 50)	*p* value
Sex (femeal/male)	16/14	15/15	0.82
Age (years)	44.86 ± 9.48	45.86 ± 10.55	0.16
Smoker/non-smoker	12/18	13/17	0.67
Protein (g/day)	80.34 ± 12.23	81.21 ± 11.13	0.56
Fat (g/day)	71.45 ± 21.27	72.10 ± 22.09	0.39
Carbohydrate (g/day)	324.01 ± 108.17	325.07 ± 109.21	0.68
BMI (kg/m^2^)	22.24 ± 1.66	24.61 ± 5.30	<0.001
TC (mmmoL/L)	4.16 ± 0.51	4.59 ± 1.24	<0.001
TG (mmmoL/L)	0.90 ± 0.32	1.59 ± 0.53	<0.001
Fasting glucose (mmmoL/L)	4.03 ± 0.47	5.69 ± 0.42	<0.001
2 h-glucose (mmmoL/L)	4.67 ± 0.99	6.13 ± 1.14	<0.001
SBP (mmHg)	112.70 ± 6.43	136.62 ± 15.40	<0.001
DBP (mmHg)	75.07 ± 6.02	80.16 ± 9.44	<0.001
HAc1 (%)	5.2 ± 0.2	6.2 ± 0.3	0.008
Fasting insulin (mU/L)	6.07 ± 2.12	7.19 ± 2.42	0.02
2 h-insulin (mU/L)	8.55 ± 2.08	23.45 ± 2.5	<0.001
HOMR-IR	1.09 ± 0.46	1.90 ± 0.46	<0.001

SBP: Systolic blood pressure; DBP: Diastolic blood pressure; TG: Triglycerides; TC: Total cholesterol. FBG: Fasting plasma glucose; 2 h-PG: 2 h Postprandial plasma glucose.

### 2.2. Study 2: Postprandial Amino Acid and Biogenic Amine Profiles after Three Test Loads in IFG Individuals

IFG individuals in study 1 were given glucose (GL), WR and HB test loads. The WR load was white rice (Qizheng Company, Lanzhou, China) and the HB load was highland barley (Qizheng Company, Lanzhou, China). Glucose was purchased from the pharmacy (Harbin Medical University, Harbin, China). WR and HB loads were boiled before they were served. The amounts of WR load (89.7 g) and HB load (100.2 g) were calculated to provide the total energy available from 75 g glucose. Details of the test loads are given in Supplementary Table S1. Participants were given a test load at 9 A.M. (breakfast) and were required to consume it within 20 min. Blood samples were collected at time zero (9 A.M.) and at 30, 60, 90 and 120 min later, centrifuged immediately at 3000× *g* for 15 min at room temperature and the supernatant was stored at –80 °C.

Each test day was separated by a washout period of seven days. All participants were asked to avoid heavy exercise and intake of alcohol 24 h before each test day. The day before each test day, participants were provided with a standardized meal and snack, which contained 15% (*w*/*v*) protein, 50% (*w*/*v*) carbohydrate and 35% (*w*/*v*) fat. The subjects were instructed to eat and drink the same prescribed foods at 8 A.M. on each test day.

### 2.3. Biochemical Measurements

Serum glucose, TC, low-density lipoprotein cholesterol (LDL-c), high-density lipoprotein cholesterol (HDL-c) and TGs were measured with kits purchased from Biosino Biotechnology (Beijing, China), standard enzymatic colorimetric techniques and with an auto-analyzer (MOL-300, Beijing, China). Hemoglobin A1c (HbA1c) was measured by a hemoglobin A1c analyzer on a BX5DS5Menarini-ArkrayKDKHA8140 (Arkray, Kyoto, Japan). Serum insulin was measured with an auto-analyzer using commercial kits (Centaur, Bayer Corporation, Bayer Leverkusen, Germany).

### 2.4. Serum Preparation

Serum amino acids and biogenic amines were prepared as described [16]. Briefly, each serum sample (50 μL) was used for metabolite extraction before UPLC-TQ-MS analysis. The metabolite extraction procedure was carried out after adding 250 μL of acetonitrile/methanol/formic acid (74.9:24.9:0.2 by vol.) containing two additional stable isotope-labeled internal standards for valine-d8 and phenylalanine-d8 in serum. After vortex mixing for 1 min, the mixture was kept at room temperature for 10 min and then centrifuged at 14,000× *g* for 10 min at 4 °C. The solution was filtered through a syringe filter (0.22 μm pore size) then placed into a sampling vial for UPLC-TQ-MS analysis.

### 2.5. UPLC-TQ-MS Analysis

UPLC-TQ-MS analysis was done with a Waters ACQUITY UPLC system (Waters Corporation, Milford, MA, USA) coupled to a Waters Xevo TQD mass spectrometer (Waters Corporation, Manchester, UK). A portion (2 μL) of the sample solution was injected into an ACQUITY UPLC™ HILIC column (100 mm × 2.1 mm *i.d.*, 1.7 μm film thickness; Waters Corporation, Milford, MA, USA). The flow rate of the mobile phase was 300 μL/min. Analytes were recovered from the column by gradient elution using solution A (10 mM aqueous ammonium formate, 0.1% (*v*/*v*) formic acid) and solution B (0.1% (*v*/*v*) formic acid in acetonitrile). The optimized conditions for the UPLC separation and ESI-TQ-MS detection are given in Supplementary Table S2.

MS analyses used electrospray ionization (ESI) and multiple reaction monitoring scans in the positive ion mode. Cone voltage and collision energies for 30 ms were optimized for each transition, the ion spray voltage was 3.2 kV and the source temperature was 150 °C. Internal standard peak areas were monitored for quality control and individual samples with peak areas differing from the group mean by more than two standard deviations were reanalyzed. MarkerLynx Application Manager software (version 4.1; Waters Corporation, Milford, MA, USA) was used for automated peak integration and metabolite peaks were reviewed manually for quality of integration and compared against a known standard to confirm identity.

### 2.6. Statistical Analysis

Values are presented as mean ± SD. Statistical analyses were done with SPSS 13.0 software (SPSS, Chicago, IL, USA). *p* ≤ 0.05 was considered statistically significant. The area under the curve (AUC) was calculated using the trapezoidal rule to quantify overall response to different loads, which reflected both the amount and duration of the response. Significance was determined by the two-tailed Student’s *t* test, analysis of covariance (ANCOVA) and repeated-measures ANOVA followed by the Tukey *post hoc* test.

Amino acid and biogenic amine variables between IFG individuals and controls were analyzed by ANCOVA adjusting for potential covariates (BMI, TG, TC, 2 h-PG, blood pressure and insulin level). The time course of glucose, insulin and postprandial amino acid and biogenic amine responses were analyzed by repeated-measures ANOVA followed by a Tukey *post hoc* test. Differences in the postprandial response between time and different loads were assessed via time × test load interaction tests. The correlation of postprandial changes in amino acids, fasting glucose and insulin, 2 h glucose and insulin, and insulin secretory indices during three test loads was assessed using Pearson’s correlation test.

## 3. Results

### 3.1. Study 1: The Amino Acid and Biogenic Amine Profiles Change in the Control and IFG Participants during 120 min OGTT

In the fasting state, 30 amino acids and biogenic amines were detected in all participants (Supplementary Table S3). The levels of 14 metabolites changed significantly in the IFG group compared to the controls. Compared with the control, ten metabolites (leucine, valine, isoleucine, phenylalanine, alanine, creatinine, glutamic acid, aminobutyric acid, cotinine and l-α-glycerophosphorylcholine, increased significantly (*p* < 0.05) and the levels of four metabolites (methionine, γ-aminobutyric acid (GABA), asparagine and allantoin) decreased.

**Figure 1:**
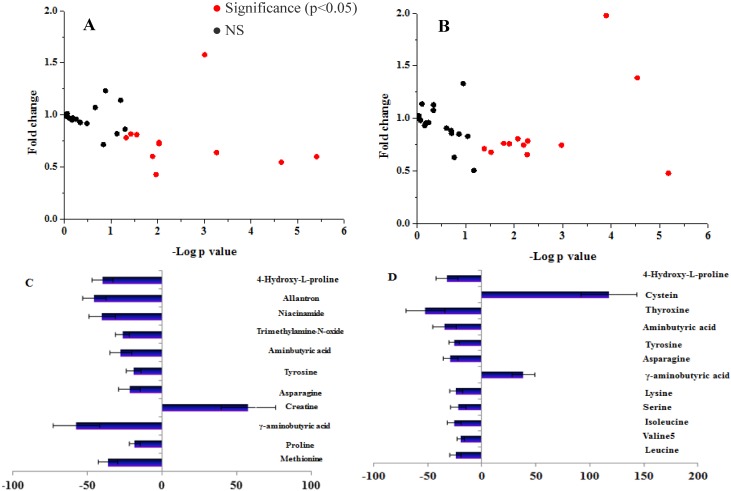
Fold change and significance of metabolite change during an oral glucose challenge in the control (**A**) and the IFG (**B**) groups, and significant percent change of metabolites from fasting to 2-h samples during an OGTT in control (**C**) and the IFG (**D**) groups. Dots represent the 30 metabolites detected in serum. Significant (*p* < 0.05) changes are colored red. Fold changes and percent changes for the metabolites (X) detected by UPLC-TQ-MS in three loads were calculated as follows: X _Fold change_ = (X _Concentration at different time (30, 60, 90, 120 min)_/X _Concentration at baseline_); X _Percent change_ = (X _Concentration at different time (30, 60, 90, 120 min)_ − X _Concentration at baseline_)/(X _Concentration at baseline_).

In both control and IFG participants, 18 metabolites were unaltered in the postprandial state compared to baseline levels (Figure 1). Levels of 12 metabolites in IFG individuals and 11 metabolites in controls were altered significantly (*p* < 0.05) according to the OGTT (Figure 1C,D). In the controls, ten metabolites decreased and only creatine increased compared to the fasting state (Figure 1C). Ten metabolites were decreased at 120 min in IFG individuals compared to the fasting state, whereas GABA and cysteine were increased (Figure 1D). GABA was the important metabolite with different dynamic change during OGTT between the IFG and control groups. Postprandial GABA was increased (fold change 1.39 ± 0.45) at 120 min in IFG participants and was decreased significantly in the controls (fold change 0.43 ± 0.18).

### 3.2. Study 2: The Postprandial Amino Acid and Biogenic Amine Profiles after Three Test Loads in the IFG Group

#### 3.2.1. Glucose and Insulin Profiles after Three Test Loads

**Figure 2:**
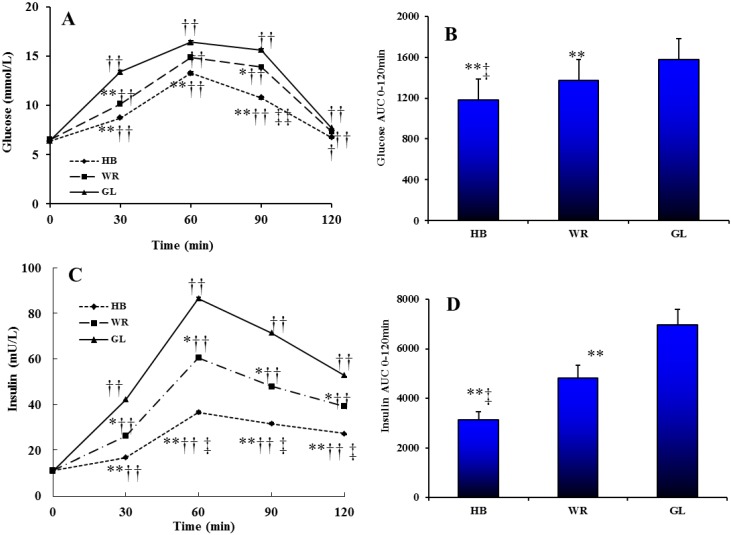
The changes in serum glucose level (**A**) and insulin level (**C**) during tree test loads. The AUC of glucose (**B**) and insulin (**D**) between 0 and 120 min of different test loads. Triangle, glucose load (GL); box, WR load (WR); diamond, HB load (HB). Repeated-measures ANOVA showed significant main effects of test load, time or a time × test load interaction on glucose (test load, *p* < 0.001; time, *p* < 0.001; interaction, *p* = 0.002) and insulin (test load, *p* < 0.001; time, *p* < 0.001; interaction, *p* < 0.001). * *p* < 0.05, ** *p* < 0.01, HB or WR *vs.* GL at the same time point using repeated measures ANOVA analysis with a Tukey *post hoc* test. ‡ *p* < 0.05, ‡‡ *p* < 0.01, HB *vs.* WR at the same time point of the ingesting WR using repeated measures ANOVA analysis with LSD *post hoc* test. † *p* < 0.05, †† *p* < 0.01, compared with the baseline in the same treated group using multiple comparisons analysis with LSD *post hoc* test.

The levels of serum glucose and insulin following different test loads showed a postprandial increase followed by a decrease (Figure 2A,C). There were significant effects of test load, time and time × test load interaction on glucose (test load *p* < 0.001; time *p* < 0.001; interaction *p* < 0.002) and insulin (test load *p* < 0.001; time *p* < 0.001; interaction *p* < 0.001). At 0–120 min the AUC for serum glucose and insulin was significantly smaller for the HB load compared to WR and GL loads (Figure 2B,D).

#### 3.2.2. The Postprandial Amino Acid and Biogenic Amine Profiles after Three Test Loads

**Figure 3:**
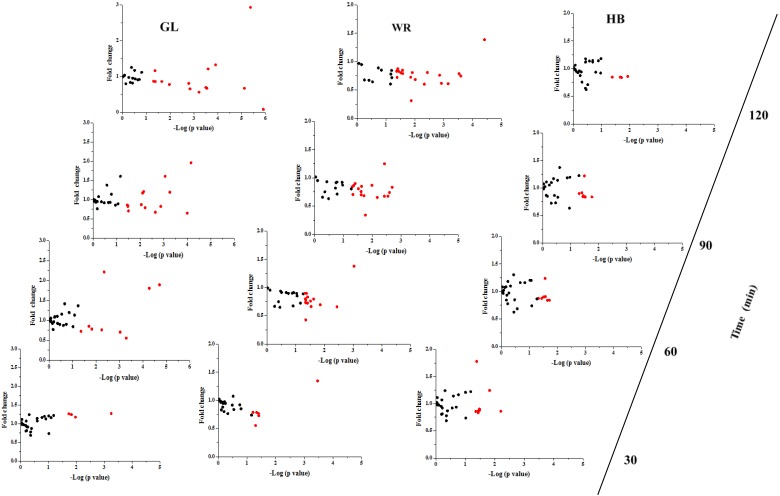
Fold change and significance of metabolite change in the IFG group during different test loads. Fold changes and percent changes for the metabolites (X) detected by UPLC-TQ-MS in three loads were calculated as follows: X _Fold change_ = (X _Concentration at different time (30, 60, 90, 120 min)_/X _Concentration at baseline_); X _Percent change_ = (X _Concentration at different time (30, 60, 90, 120 min)_ − X _Concentration at baseline_)/(X _Concentration at baseline_).

**Figure 4:**
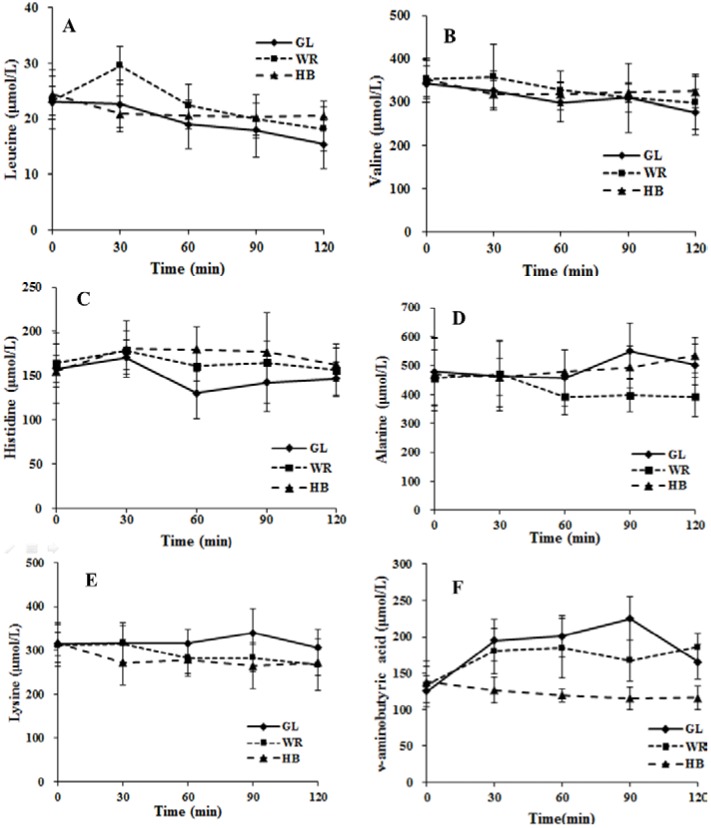
The changes in serum leucine (**A**), valine (**B**), histidine (**C**), alanine (**D**), lysine (**E**) and ν-aminobutyric acid (**F**) during three test loads. Diamond, glucose load (GL); box, WR load (WR); Triangle, HB load (HB). Repeated-measures ANOVA showed significant main effects of test load, time or a time × test load interaction on leucine (test load, *p* = 0.041; time, *p* < 0.001; interaction, *p* = 0.005), valine (test load, *p* = 0.199; time, *p* = 0.001; interaction, *p* = 0.013), histidine (test load, *p* = 0.008; time, *p* < 0.001; interaction, *p* < 0.001), alanine (test load, *p* = 0.009; time, *p* = 0.297; interaction, *p* = 0.005), lysine (test load, *p* = 0.016; time, *p* = 0.013; interaction, *p* = 0.028), ν-aminobutyric acid (test load, *p* < 0.001; time, *p* < 0.001; interaction, *p* < 0.001).

The fold change and significance of metabolite changes during three test loads in IFG participants are shown in Figure 3 and Supplementary Figures S2–S4. The number of significant metabolites increased from time zero–120 min for GL and WR loads, whereas the number of significant metabolites was not altered from time zero–90 min and decreased at 120 min during the HB load. With regard to the change of metabolites at 120 min, the levels of 15 metabolites after a GL load (Supplementary Figure S2) and 20 metabolites after a WR load (Supplementary Figure S3) were changed significantly and only four metabolites decreased significantly after a HB load (Supplementary Figure S4). The changed levels of GABA were different among the three test loads. The postprandial level of GABA was higher after GL and WR loads but was not altered significantly at 30, 60 or 90 min and was decreased significantly (−15.5%) at 120 min for a HB load. The mean concentrations of GABA after a HB load were significantly lower (*p* < 0.05) at 30, 60, 90 and 120 min compared to GL and WR loads (Figure 4F). For the postprandial change in GABA after the three test loads, there were significant differences (*p* < 0.001) in the time, test load and the interaction of time × test load (Figure 4). The 0–120 min AUC for GABA after an HB load was significantly lower (*p* < 0.05) compared to GL and WR loads (Supplementary Figure S5F).

#### 3.2.3. Correlation between Postprandial Metabolites, Glucose and Insulin Following Different Loads

Postprandial changes in the concentrations of leucine, histidine and lysine during the three test loads were correlated with 2h-insulin (Table 2), whereas the postprandial change of alanine was correlated negatively (*p* < 0.05). GABA was associated positively with 2 h-glucose during three test loads with 2 h-glucose and fasting insulin in the GL and WR loads, while GABA was related negatively to 2 h-glucose in the HB load. The postprandial dynamic change in six significant metabolites correlated with glucose and insulin during the three test loads is shown in Figure 4 and Supplementary Figure S5. For the postprandial change in six significant metabolites, there was a significant difference (*p* < 0.05) in the interaction of time × test load (Figure 4). AUC for leucine, valine and histidine for the HB load was significantly greater (*p* < 0.05) compared to the GL load and AUC for lysine and GABA was significantly smaller (*p* < 0.05) compared to the GL load.

**Table 2:** Associations between glucose, insulin and insulin sensitivity index and percent change of metabolites from fasting to 2-h sample response to three test loads.

Metabolites		GL		
	Fasting Glucose	2 h-glucose	Fasting Insulin	2 h-Insulin
Leucine				−0.647 (0.024)
Valine				
Histidine				−0.604 (0.029)
Alanine		−0.600 (0.048)		
Lysine				−0.587 (0.034)
γ-aminobutyric acid		0.621 (0.023)	0.551 (0.040)	
		HB		
	Fasting glucose	2 h-glucose	Fasting insulin	2 h-insulin
Leucine				−0.774 (0.023)
Valine				
Histidine				−0.637 (0.045)
Alanine		−0.607 (0.041)		
Lysine				−0.548 (0.042)
γ-aminobutyric acid		0.614 (0.038)	0.514 (0.044)	
		WR		
	Fasting glucose	2 h-glucose	Fasting insulin	2 h-insulin
Leucine				−0.627 (0.038)
Valine				
Histidine		0.691 (0.018)		−0.621 (0.029)
Alanine		−0.761 (0.006)		
Lysine				−0.568 (0.042)
γ-aminobutyric acid		−0.572 (0.041)		

Data was analyzed by Pearson correlation. Data for metabolites, glucose, insulin and insulin sensitivity index associations, which did not achieve statistical significance are not included. Data are presented as standardized correlation-coefficients (*p*-values). Standardized correlation-coefficients were computed from standard deviations with *p*-values < 0.05.

## 4. Discussion

In this study, there were different fasting amino acid and biogenic amine profiles and postprandial response of amino acid and biogenic amine profiles between control and IFG participants. Recently, the idea that BCAAs and several related amino acids are linearly related to the homeostasis model assessment of insulin resistance has been supported by the results of some studies [8,17]. Other studies demonstrated elevated levels of BCAA were associated strongly with the future risk of diabetes and valine might be a biomarker for the identification of pre-diabetes such as IFG [8,18]. Our results suggested there were higher levels of AAAs and BCAAs in the IFG group compared to the controls, suggesting a high level of BCAAs in a clinical study might imply the risk of IFG or diabetes. Moreover, our study confirmed there were different metabolic changes in postprandial amino acid and biogenic amine profiles in IFG individuals during three test loads. The postprandial changes of glucose and insulin were lower after a HB load compared to GL and WR loads. Especially, there was a smaller AUC for GABA after a HB load compared to GL and WR loads. These results suggested a HB load lowered the response of insulin and further altered postprandial amino acid and biogenic amine profiles.

With regard to the regulation of the postprandial response of insulin production to a HB load, it is reported that oats and barley can decrease the insulin response because they contain β-glucan [19]. Our results demonstrated the HB load might decrease the postprandial response of insulin. The high β-glucan content (6.42%, *w*/*w*) of the HB load might be the main reason for reduction of the postprandial insulin response in this study. A potential mechanism might be related to changes in gut hormones, including GLP-1. Some studies demonstrated plasma insulin responses were associated closely with GLP-1 [20,21] and the secretion of GLP-1 is known to be influenced by diet [22]. A recent study, as well as our unpublished observations, showed there was a low level of GLP-1 during an HB load. Therefore, a low level of postprandial insulin might be attributed, at least in part, to a decrease of GLP-1 associated with consumption of a carbohydrate load containing a high content of β-glucan.

Insulin is associated closely with amino acid metabolism in the postprandial state [12]. Earlier studies showed acute hyperglycemia after a glucose load resulted in change of the amino acids profiles in healthy adults, obese individuals and impaired glucose tolerance individuals because the metabolism in the body was regulated by the postprandial level of insulin [12,23]. In agreement with these studies, there was postprandial change of amino acid and biogenic amine profiles in the IFG participants and controls in the present study. There were different metabolic alterations after the three test loads in IFG participants and the number of metabolites changed by a HB load was lower compared to GL and WR loads. The HB load contained a high level (6.42%, *w*/*w*) of β-glucan, which has been reported to prolong the postprandial insulinemic response, leading to a decreased level of postprandial insulin [24]. Therefore, the low level of insulin during a HB load in this study probably inhibited the postprandial change of some metabolites. Moreover, our data demonstrated that the change in postprandial BCAAs (leucine and valine) was correlated with insulin. BCAAs, essential amino acids for humans, have central roles in protein metabolism [25], improving glucose metabolism [26] and regulating leptin secretion during food intake [27]. Some reports have shown a gradual decrease of BCAAs during an OGTT in healthy and the impaired glucose tolerance subjects [12,23] in accord with this study. Furthermore, there were smaller decreases of these amino acids associated with an HB load compared to GL and WR loads. BCAAs are particularly sensitive to insulin action and their metabolism has been observed to be altered profoundly in insulin-resistant states. Recently, Newgard *et al.* [8] observed associations between BCAAs and insulin resistance and Tai *et al.* [17] reported insulin resistance was associated with leucine/isoleucine. DeFronzo *et al.* suggested first-phase insulin secretion is important for the inhibition of endogenous glucose production during an OGTT or a meal [28], so the less severe decrease of BCAAs during a HB load might be due to already decreased levels of postprandial insulin and the increase in early-phase insulin secretion in HB load would be expected to increase hepatic glucose production and suppress the excessive rise in plasma glucose. In short, our results suggested these postprandial changes in several amino acids, especially BCAAs, could shed new light on postprandial metabolic dysregulation in IFG individuals and HB might be helpful for controlling such dysregulation.

Another important finding of this study was the less pronounced increase of GABA during an OGTT in IFG individuals, and that the change in GABA was different among the three test loads (Figure 4). There were higher postprandial levels of GABA after GL and WR loads, whereas postprandial levels of GABA decreased significantly (−15.5%) at 120 min for a HB load. In the pancreas, GABA is produced primarily by insulin-secreting beta cells [29] and the release of GABA from these cells appears to be regulated by glucose [30]. Therefore, different levels of glucose in the three test loads might contribute to the different postprandial changes in GABA levels. Moreover, GABA had antioxidative effects [31] and acute hyperglycemia after a meal or glucose load increases oxidative stress [32]. Thus, increased GABA with GL and WR loads might be a stress response to ameliorate or prevent the high postprandial oxidative stress in IFG individuals. In summary, different postprandial GABA among the three test loads indicated GABA was regulated by diet.

This study has several limitations: Firstly, the duration of the postprandial investigation was short (only 2 h), thus, it is relevant only to the short-term effects following a carbohydrate load. Secondly, the effect of different proteins in the WR and HB loads on the postprandial amino acid and biogenic amine profiles was not considered. Finally, the number of participants was small and care must be exercised in the extrapolation of our findings to larger populations. Therefore, further studies are needed in this area.

## 5. Conclusions

In conclusion, our study found the amino acid and biogenic amine profiles were different between IFG individuals and controls at baseline and after an OGTT. This study showed also that a HB load was associated with a low postprandial glucose and insulin response, which resulted in changes of the amino acid and biogenic amine profiles. The results of this study offer new insights into the complex physiological regulation of metabolism in IFG individuals during the consumption of different sources of dietary carbohydrate.

## Supplementary Files

Supplementary File 1
