# Supplementary material for: Postprandial Differences in the Amino Acid and Biogenic Amines Profiles of Impaired Fasting Glucose Individuals after Intake of Highland Barley
Source: Nutrients. 2015 Jul 9;7(7):5556–71. doi: 10.3390/nu7075238 (PMC4517015; doi:10.3390/nu7075238)
Supplement: Supplementary File 1 [file nutrients-07-05238-s001.doc]

**Supplementary Information**

**Table S1.** The characteristics of three test loads.

|  | **GL Load** | **WR Load** | **HB Load** |
| --- | --- | --- | --- |
| Energy each load |  |  |  |
| Kal | 307.5 | 307.5 | 307.5 |
| KJ | 1286 | 1286 | 1286 |
| Total mass (g/test load) | 75 | 89.7 | 100.2 |
| Macronutrient composition |  |  |  |
| Protein (g) | - | 7.4 | 10.0 |
| Fat (g) | - | 0.8 | 1.0 |
| Carbohydrate (g) | 75 | 76.5 | 64.5 |
| β-glucan(g) | - | 0.75 | 6.42 |
| Insoluble dietary fiber (g) | - | 0.7 | 8.7 |
| GI value | 100 | 83 ± 5 | 35 ± 4 |

**Table S2.** UPLC-ESI-TQ-MS Conditions.

| **UPLC (Separation Conditions)** | |
| --- | --- |
| Column | ACQUITY UPLC™ HILIC column |
|  | (100 mm × 2.1 mm *i.d.*, 1.7 μm, Waters) |
| Mobile phase A | 10 mM ammonium formate and 0.1% formic acid, *v*/*v* |
| Mobile phase B | Acetonitrile with 0.1% formic acid, *v*/*v* |
| Gradient elution | A% = 5% maintained (0–0.5 min), increased to 40% in 6 min |
|  | 50% linearly increased (6–7 min) |
|  | 50% maintained 1 min(7–8 min), followed by re-equilibration |
|  | to the initial conditions in 6 min (8–15 min) |
| Flow rate | 0.30 mL/min |
| Injection volume | 2 μL |
| TQ-MS Condition | |
| Polarity | ESI positive |
| Capillary voltage | 3200 V |
| Desolation gas flow | 650 L/h |
| Cone gas flow | 50 L/h |
| Source temperature | 150 °C |
| Desolvation temperature | 400 °C |

**Table S3.** Quantitative analysis of amino acids, biogenic amines and fatty acids in the fasting serum between the control and IFG groups.

| **Metabolites (μmoL/L)** | **Control (*n* = 50)** | **IFG (*n* = 50)** | ***p* value** |
| --- | --- | --- | --- |
| Threonine | 169.51 ± 52.67 | 181.85 ± 42.33 | 0.378 |
| Leucine | 21.88 ± 7.41 | 27.10 ± 6.17 | 0.011 |
| Arginine | 141.33 ± 660.52 | 169.45 ± 411.73 | 0.094 |
| Valine | 298.69 ± 84.51 | 343.75 ± 64.23 | 0.045 |
| Isoleucine | 35.24 ± 9.32 | 41.09 ± 8.49 | 0.026 |
| Phenylalanine | 122.54 ± 28.73 | 139.16 ± 27.83 | 0.045 |
| Tryptophan | 71.91 ± 15.68 | 78.66 ± 16.50 | 0.144 |

***Table S3.*** *Cont.*

| **Metabolites (μmoL/L)** | **Control (*n* = 50)** | **IFG (*n* = 50)** | ***p* value** |
| --- | --- | --- | --- |
| Serine | 173.40 ± 42.26 | 193.32 ± 41.04 | 0.100 |
| Methionine | 15.10 ± 0.04 | 10.06 ± 0.03 | 0.001 |
| Glycine | 362.53 ± 121.45 | 314.98 ± 66.27 | 0.110 |
| Proline | 215.59 ± 77.30 | 221.78 ± 55.82 | 0.755 |
| Histidine | 131.42 ± 45.83 | 133.91 ± 35.69 | 0.836 |
| Alanine | 536.85 ± 188.81 | 665.48 ± 157.90 | 0.014 |
| Lysine | 450.73 ± 156.70 | 439.44 ± 129.19 | 0.787 |
| γ-aminobutyric acid | 323.96 ± 101.84 | 127.61 ± 17.86 | <0.001 |
| Creatinine | 87.31 ± 25.80 | 103.86 ± 23.06 | 0.023 |
| Dimethylglycine | 11.74 ± 4.85 | 11.23 ± 6.75 | 0.755 |
| Creatine | 13.52 ± 7.38 | 10.71 ± 5.16 | 0.139 |
| Glutamic acid | 39.13 ± 13.25 | 44.49 ± 14.35 | 0.016 |
| Asparagine | 17.09 ± 8.60 | 15.57 ± 7.44 | 0.515 |
| Tyrosine | 87.92 ± 28.86 | 105.22 ± 22.63 | 0.026 |
| Thyroxine | 12.22 ± 6.10 | 13.91 ± 6.44 | 0.344 |
| Aminbutyric acid | 0.023 ± 0.01 | 0.05 ± 0.02 | <0.001 |
| Trimethylamine-N-oxide | 0.37 ± 0.17 | 0.57 ± 0.23 | 0.118 |
| Niacinamide | 0.27 ± 0.10 | 0.38 ± 0.21 | 0.214 |
| Cystein | 0.20 ± 0.08 | 0.16 ± 0.05 | 0.084 |
| Cotinine | 0.029 ± 0.01 | 0.54 ± 0.15 | 0.001 |
| Allantoin | 19.19 ± 9.61 | 10.41 ± 5.17 | <0.001 |
| 4-Hydroxy-L-proline | 16.34 ± 6.09 | 16.64 ± 8.63 | 0.928 |
| L-α-Glycerophosphorylcholine | 13.39 ± 4.19 | 16.55 ± 4.72 | 0.015 |


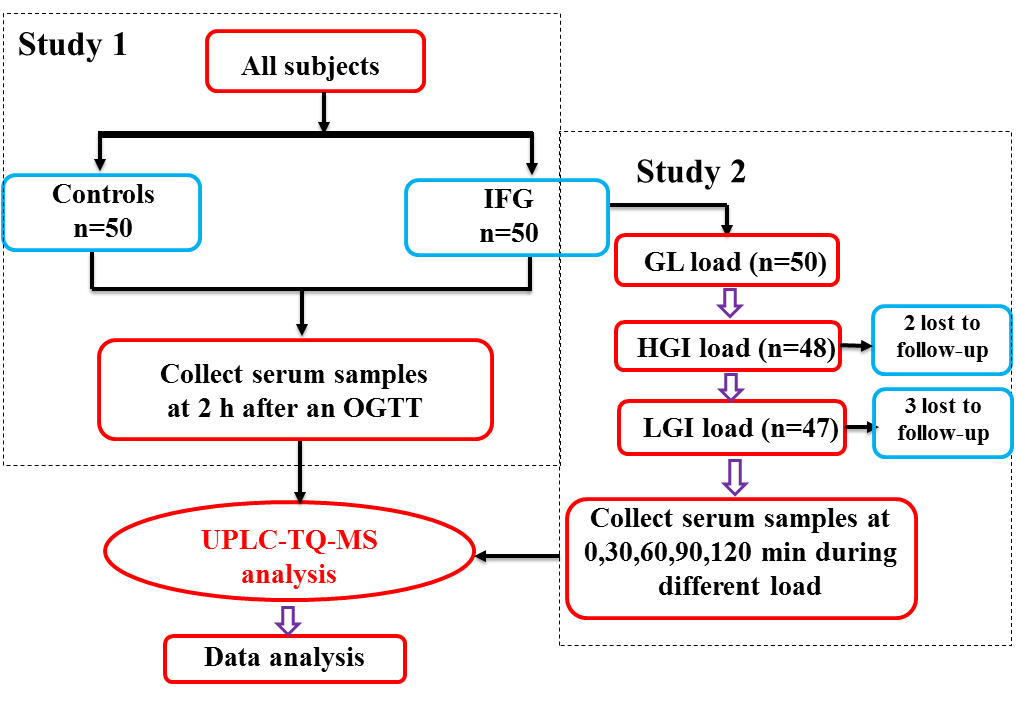


**Figure S1.** The design of this study.

| 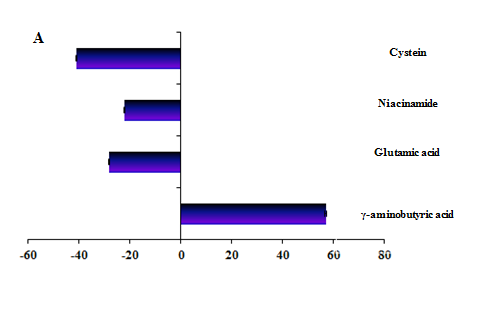 | 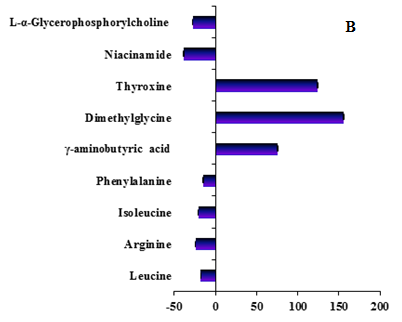 |
| --- | --- |
| 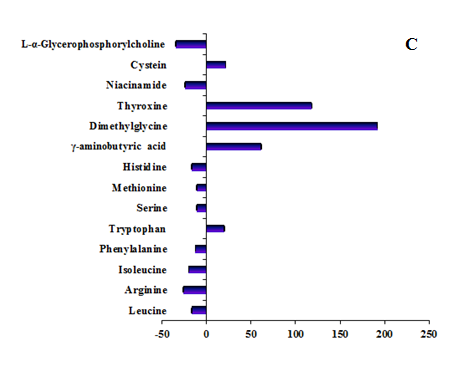 | 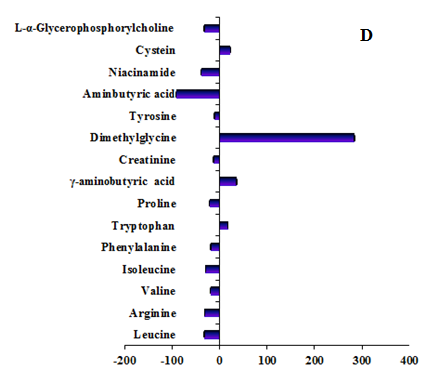 |

**Figure S2.** Percent change of metabolites from fasting to 2-h samples during the GL load in the IFG subjects. (**A**): 30 min; (**B**): 60 min; (**C**): 90 min, (**D**): 120 min. Percent changes for the metabolites (X) detected by UPLC-TQ-MS in three loads were calculated as follows: X _Percent change_ = (X Concentration at different time (30, 60, 90, 120 min) − X _Concentration at baseline_)/(X _Concentration at baseline_).

| 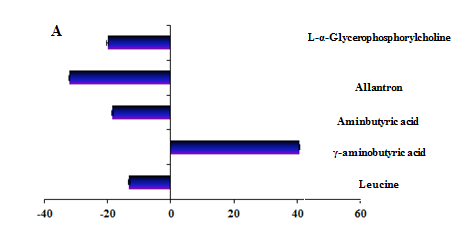 | 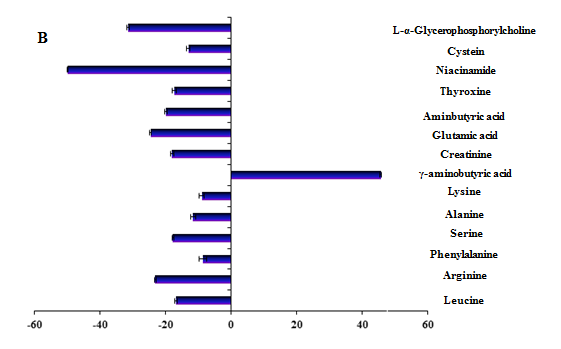 |
| --- | --- |

**Figure S3.** *Cont.*

| 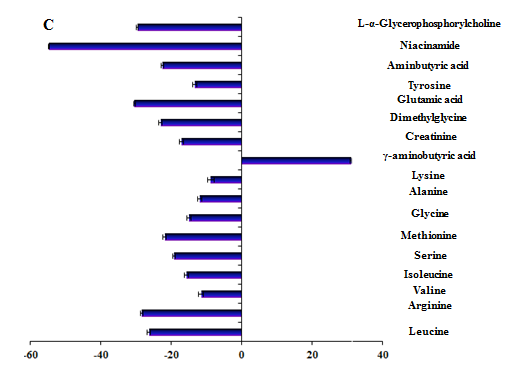 | 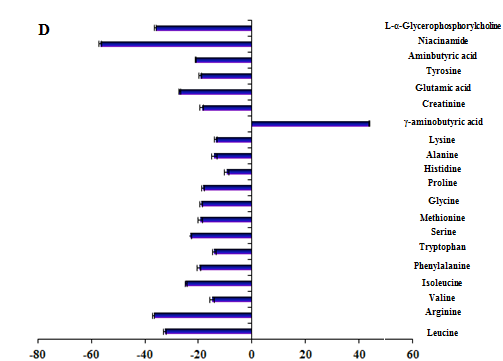 |
| --- | --- |

**Figure S3.** Percent change of metabolites from fasting to 2-h samples during the WR load in the IFG subjects. (**A**): 30 min; (**B**): 60 min; (**C**): 90 min, (**D**): 120 min. Percent changes for the metabolites (X) detected by UPLC-TQ-MS in three loads were calculated as follows: X _Percent change_ = (X Concentration at different time (30, 60, 90, 120 min) − X _Concentration at baseline_)/(X _Concentration at baseline_).

| 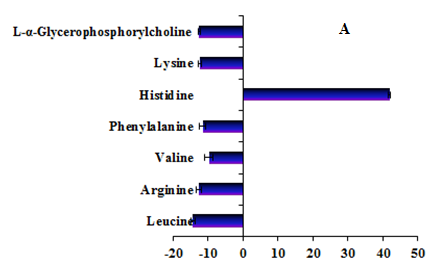 | 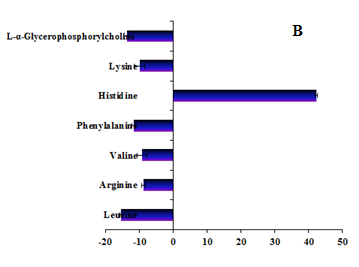 |
| --- | --- |
| 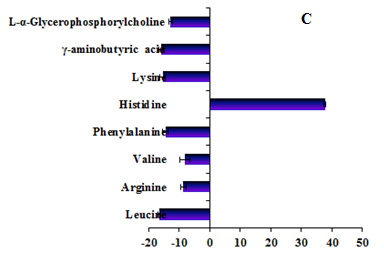 | 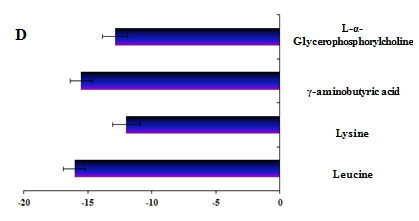 |

**Figure S4.** Percent change of metabolites from fasting to 2-h samples during the WR load in the IFG subjects. (**A**): 30 min; (**B**): 60 min; (**C**): 90 min, (**D**): 120 min. Percent changes for the metabolites (X) detected by UPLC-TQ-MS in three loads were calculated as follows: X _Percent change_ = (X Concentration at different time (30, 60, 90, 120 min) − X _Concentration at baseline_)/(X _Concentration at baseline_).

| 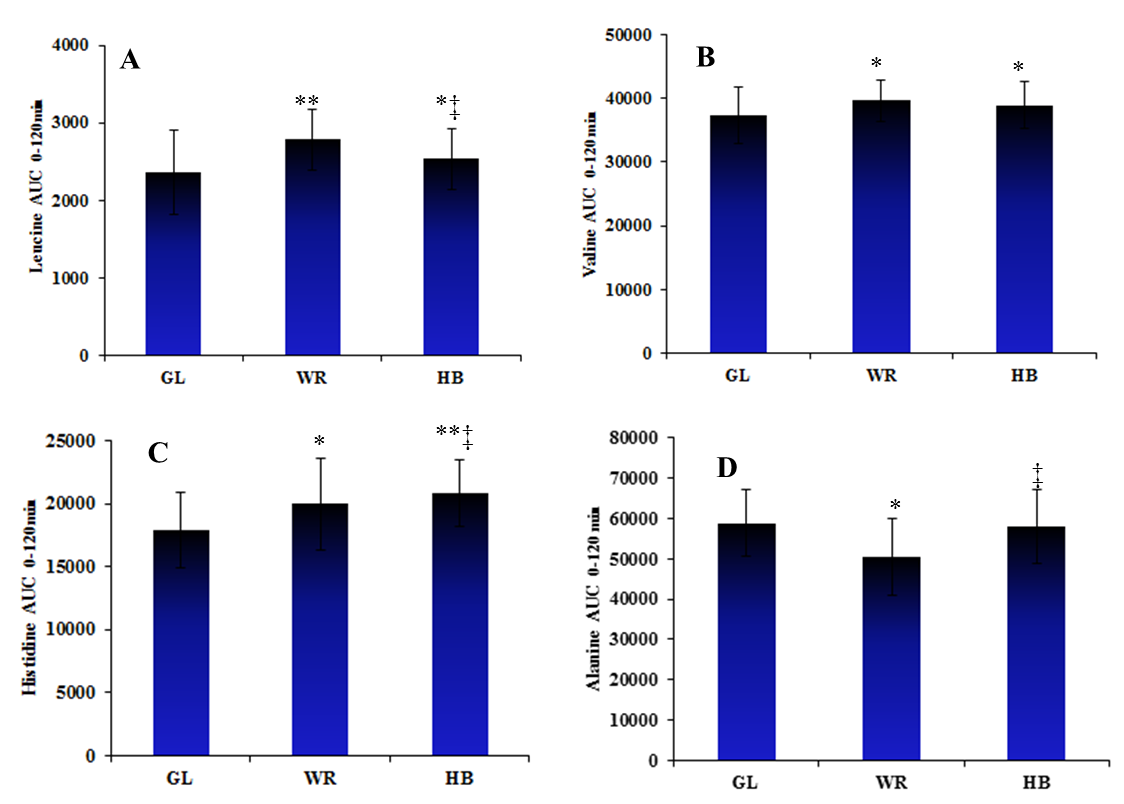 |
| --- |
| 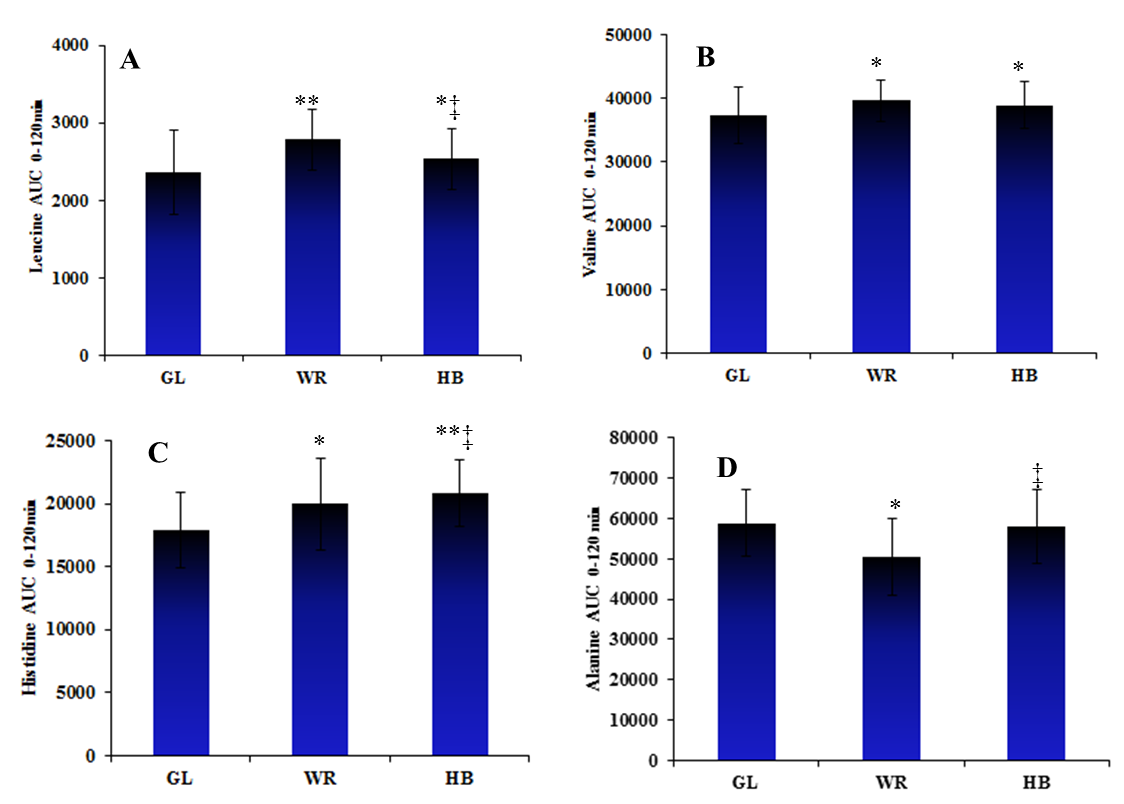 |
| 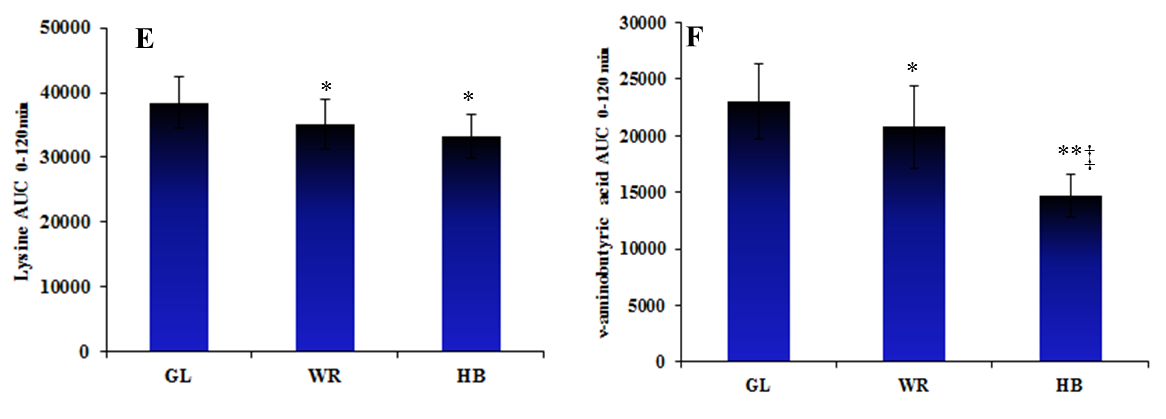 |

**Figure S5.** The AUC of leucine (**A**), valine (**B**), histidine (**C**), alanine (**D**), lysine (**E**) and ν-aminobutyric acid (**F**) between 0 and 120 min of different test loads. * *p* < 0.05, ** *p* < 0.01, HB or WR *vs.* GL. ^‡^ *p* < 0.05, HB *vs.* WR.

© 2015 by the authors; licensee MDPI, Basel, Switzerland. This article is an open access article distributed under the terms and conditions of the Creative Commons Attribution license (http://creativecommons.org/licenses/by/4.0/).
